# A Diverse Range of Novel RNA Viruses in Geographically Distinct Honey Bee Populations

**DOI:** 10.1128/JVI.00158-17

**Published:** 2017-07-27

**Authors:** Emily J. Remnant, Mang Shi, Gabriele Buchmann, Tjeerd Blacquière, Edward C. Holmes, Madeleine Beekman, Alyson Ashe

**Affiliations:** a Behaviour and Genetics of Social Insects Laboratory, School of Life and Environmental Sciences, The University of Sydney, Sydney, Australia; b Marie Bashir Institute for Infectious Diseases and Biosecurity, Sydney Medical School, The University of Sydney, Sydney, Australia; c Charles Perkins Centre, The University of Sydney, Sydney, Australia; d School of Life and Environmental Sciences, The University of Sydney, Sydney, Australia; e Wageningen University & Research, Wageningen, The Netherlands; University of Illinois at Chicago

**Keywords:** RNA interference, arthropod vectors, insect viruses, metagenomics, negative-strand RNA virus, phylogenetic analysis, plus-strand RNA virus

## Abstract

Understanding the diversity and consequences of viruses present in honey bees is critical for maintaining pollinator health and managing the spread of disease. The viral landscape of honey bees (Apis mellifera) has changed dramatically since the emergence of the parasitic mite Varroa destructor, which increased the spread of virulent variants of viruses such as deformed wing virus. Previous genomic studies have focused on colonies suffering from infections by Varroa and virulent viruses, which could mask other viral species present in honey bees, resulting in a distorted view of viral diversity. To capture the viral diversity within colonies that are exposed to mites but do not suffer the ultimate consequences of the infestation, we examined populations of honey bees that have evolved naturally or have been selected for resistance to Varroa. This analysis revealed seven novel viruses isolated from honey bees sampled globally, including the first identification of negative-sense RNA viruses in honey bees. Notably, two rhabdoviruses were present in three geographically diverse locations and were also present in Varroa mites parasitizing the bees. To characterize the antiviral response, we performed deep sequencing of small RNA populations in honey bees and mites. This provided evidence of a Dicer-mediated immune response in honey bees, while the viral small RNA profile in Varroa mites was novel and distinct from the response observed in bees. Overall, we show that viral diversity in honey bee colonies is greater than previously thought, which encourages additional studies of the bee virome on a global scale and which may ultimately improve disease management.

**IMPORTANCE** Honey bee populations have become increasingly susceptible to colony losses due to pathogenic viruses spread by parasitic Varroa mites. To date, 24 viruses have been described in honey bees, with most belonging to the order Picornavirales. Collapsing Varroa-infected colonies are often overwhelmed with high levels of picornaviruses. To examine the underlying viral diversity in honey bees, we employed viral metatranscriptomics analyses on three geographically diverse Varroa-resistant populations from Europe, Africa, and the Pacific. We describe seven novel viruses from a range of diverse viral families, including two viruses that are present in all three locations. In honey bees, small RNA sequences indicate that these viruses are processed by Dicer and the RNA interference pathway, whereas Varroa mites produce strikingly novel small RNA patterns. This work increases the number and diversity of known honey bee viruses and will ultimately contribute to improved disease management in our most important agricultural pollinator.

## INTRODUCTION

Viruses are some of the most common pathogens contributing to declining honey bee health and colony losses worldwide (1–4), and at least 24 viruses have been described in the western honey bee, Apis mellifera (1, 5–12). Historically, viruses were identified based on pathological symptoms present in diseased bees by using serological methods (6, 7, 13, 14). Other than two DNA viruses (10, 11, 15), all currently characterized honey bee virus genomes comprise positive-sense, single-stranded RNA molecules (4, 16–19). Indeed, most viruses from honey bees fall into two families within the order Picornavirales (12, 16–21): the iflaviruses, including Sacbrood virus (SBV) and Deformed wing virus (DWV) (22, 23), and the dicistroviruses, including Black queen cell virus (BQCV) and Acute bee paralysis virus (ABPV) (24). More recently, genomic methods have identified additional viruses (5, 9, 10), which led to the discovery of a new genetic variant of DWV (23) and the Lake Sinai virus (LSV) group (5, 25). LSVs are common and widespread in honey bees, although whether they are associated with overt disease is unknown (26).

The prevalence, distribution, and virulence of honey bee viruses seem closely associated with the ectoparasitic mite Varroa destructor (2, 3, 22–24, 27). Varroa mites can act as an important virus vector, causing a dramatic change in both the viral landscape and virulence (3, 28, 29). As Varroa mites spread globally due to the human-mediated translocation of honey bees during the early to mid-20th century, so did virulent viruses, leading to the widespread loss of managed and wild honey bee colonies (2, 3, 27, 29, 30). Viruses from the Picornavirales appear to have a particularly close association with Varroa mites (8).

While Picornavirales are commonplace, it is striking that negative-sense RNA viruses apparently seem to be absent from honey bee colonies. This is puzzling, as negative-sense viruses, such as members of the Rhabdoviridae and Bunyaviridae, are widespread in other arthropods (31–33). Similarly, other categories of positive-sense RNA viruses, such as the Flaviviridae, are also common in insects but seemingly absent from studies of honey bees performed to date (34, 35). The Flaviviridae are notable, as they include mosquito and tick vector-borne viruses responsible for a number of important human diseases, including dengue, West Nile, and Zika, as well as other insect-specific viruses (35, 36).

Honey bees exhibit multiple antiviral defense mechanisms, including one of the key innate immune responses in insects, the RNA interference (RNAi) pathway (19, 37, 38). During virus replication, double-stranded RNA (dsRNA) intermediates are formed, which are recognized and cleaved by the endonuclease enzyme Dicer into 21- to 22-nucleotide (nt) fragments called small inhibitory RNAs (siRNAs) (39). These siRNAs are then bound by Argonaute proteins, which guide the RNA-induced silencing complex (RISC) and degrade complementary RNA molecules such as viral genomes (38). Honey bees produce siRNAs that match the predominant viruses in collapsing colonies (40) and can also produce a small RNA response when experimentally infected with double-stranded RNA (41, 42).

The use of RNA sequencing in a broad range of arthropods has greatly enriched our understanding of virus biodiversity (31, 33, 35, 43–46). It therefore seems opportune to revisit the viral diversity of honey bee colonies using similarly high-powered techniques. Accordingly, we screened honey bee populations for the presence of RNA viruses using total RNA transcriptome sequencing, so-called “metatranscriptomics” (33). We specifically focused on honey bee populations that have Varroa parasites but do not appear to suffer any negative consequences (47) and populations without Varroa mites. These populations were selected to avoid the possibility that novel viruses are outcompeted by highly virulent viral strains associated with Varroa mites. Notably, we also sampled bees from geographically diverse locations in Europe, Africa, and the Pacific to determine how viral diversity varies on a spatial scale.

## RESULTS

### Viral diversity in Varroa-resistant and Varroa-free honey bee populations.

We examined the viral diversity of three Apis mellifera populations from Europe, Africa, and the Pacific by sequencing total ribosome-depleted RNA extracted from worker honey bees. We sampled honey bee colonies at the Amsterdam Water Dunes (The Netherlands), Stellenbosch and Robben Island (South Africa), and Vava'u and Tongatapu islands (Kingdom of Tonga) (Table 1). The colonies from The Netherlands are part of a selection program that started in 2008. These colonies are not treated for Varroa infestation but carry low numbers of mites (48). Colonies of Apis mellifera capensis in South Africa are similarly not treated for Varroa infestation and are naturally resistant to mites (49). The honey bee population on Robben Island became infested with Varroa mites 2 years prior to sampling, and numbers of mites per colony remain low (49). In the Pacific islands of Tonga, honey bees were most likely introduced during the 19th century, and large numbers of feral colonies are found in multiple island groups. Varroa mites were introduced to the island of Vava'u in 2006. Due to the lack of commercial beekeeping, colonies are not treated to remove mites, and, like the South African population, honey bees appear to be naturally tolerant to Varroa mites. The honey bees on Tongatapu island have never been exposed to Varroa mites but are derived from the same original source population as those on the island of Vava'u (50). We synthesized libraries from pooled RNA extracted from five individuals per colony, and 100-bp paired-end sequencing yielded a range of 4 to 9 Gb of data per library (Table 1). We assembled reads into contigs *de novo* using Trinity (51) and compared the resulting contigs to available viral protein sequences from GenBank with BLASTx.

**TABLE 1:** Data generated in this study and summary of the viruses identified

Location^*e*^	Colony	Data generated (no. of reads, data yield [Gb])^*b*^	Virus or pathogen(s) present		
			Known virus(es)	Novel virus(es)^*d*^	Other
The Netherlands, AWD	NE_AWD_1151	45,393,799, 9.08	DWV, SBV, BQCV, LSV-NE^*c*^	ADV	Nosema apis
	NE_AWD_1442	43,418,765, 8.69	DWV, SBV, BQCV	ARV-1, ARV-2	Lotmaria passim
South Africa, RI and SB	SA_RI_A	20,107,219, 4.02	DWV	ABV-1, ABV-2, ANV-1	Lotmaria passim
	SA_RI_11	20,515,230, 4.10	DWV	ABV-1, AFV-1	Lotmaria passim
	SA_RI_49	18,820,078, 3.76	DWV, SBV, BQCV, ABPV	ARV-1, ARV-2, ABV-1	Lotmaria passim
	SA_SB_C1	44,731,233, 8.95	DWV, SBV, BQCV, LSV-SA-1^*c*^		Nosema apis
	SA_SB_K2	42,022,291, 8.41	DWV, SBV, BQCV, LSV-SA-2^*c*^		Nosema apis
Tonga, V and T	T_V9	18,658,353, 3.93	DWV, SBV	ARV-1	
	T_V10	21,309,419, 4.26	DWV	ARV-1, ARV-2	
	T_T12^*a*^	21,141,746, 4.23	DWV, SBV, BQCV, LSV-TO^*c*^	ARV-1, ARV-2	
	T_T23^*a*^	19,203,423, 3.84	DWV, SBV, BQCV, LSV-TO^*c*^	ARV-1	Nosema ceranae, Leishmania sp.

a Varroa-free colonies.

b Determined by using 100-bp paired-end Illumina HiSeq sequencing.

c LSV variants are presented in Fig. S1 in the supplemental material. NE, Netherlands; SA, South Africa; TO, Tonga.

d Novel viruses are presented in Table 2 and 3.

e AWD, Amsterdam Water Dunes; RI, Robben Island; SB, Stellenbosch; V, Vava'u; T, Tongatapu.

**FIG 1:**
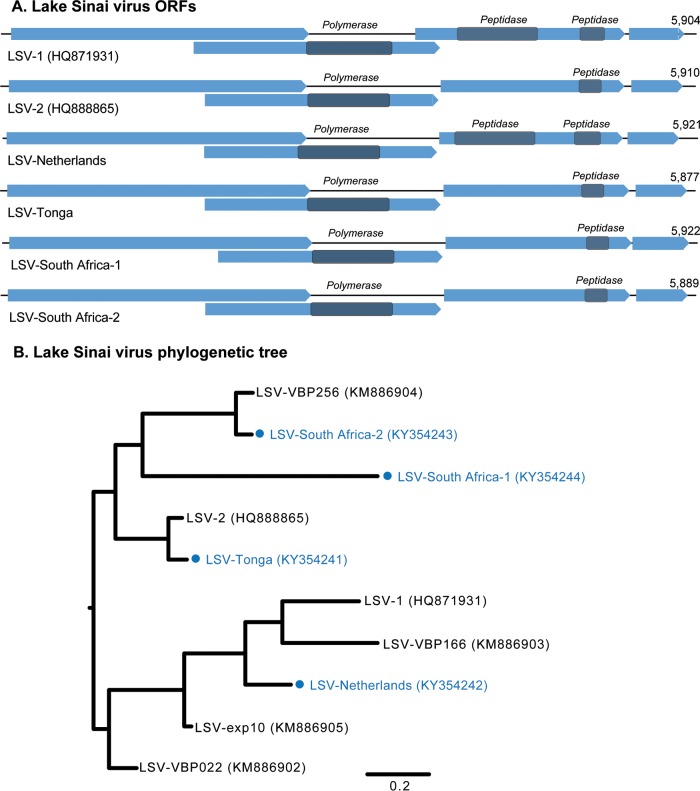
Lake Sinai virus variants identified in this study. (A) Genome structures of LSV strains identified in The Netherlands (GenBank accession number KY354242), Tonga (accession number KY354241), and South Africa (accession numbers KY354243 to KY354244), compared to previously characterized genomes of LSV-1 and LSV-2. Open reading frames are blue, and conserved functional domains are indicated (NCBI protein sequence accession numbers ARO50053 to ARO50067). (B) Maximum likelihood phylogenetic tree of nucleotide alignment of LSV strains from The Netherlands, Tonga, and South Africa with LSV-1 and -2 and other strains described previously (26).

We first examined the assembled contigs that matched previously characterized honey bee pathogens (Table 1). We found similarities in the presence and absence of known viruses in all three Varroa-resistant populations. Contigs for DWV, BQCV, and sacbrood virus (SBV) were present in all three locations. ABPV was present in one colony from Robben Island, and LSV was present in one colony each from The Netherlands and Tonga and in two colonies from South Africa (Table 1). The LSV genomes from The Netherlands, Tonga, and South Africa show 4 predicted open reading frames (ORFs), similarly to previously characterized LSV-1 and -2 genomes (Fig. 1). However, the nucleotide sequences from each location exhibit significant divergence, with 69 to 91% identity to previously characterized variants. We also examined our samples for contigs that matched common fungal, bacterial, and protozoan parasites of honey bees, such as the fungi Nosema apis and Nosema ceranae, the bacterial agents of European and American foulbrood, and the trypanosomes Crithidia mellificae and Lotmaria passim (4, 52). We observed contigs for L. passim in three colonies from Robben Island, South Africa, and one colony from The Netherlands (Table 1). The two colonies from mainland South Africa and one colony from The Netherlands contained contigs for Nosema apis, and one colony from Tonga contained contigs for Nosema ceranae, along with a single contig with similarity to Leishmania sp. (Table 1).

**FIG 2:**
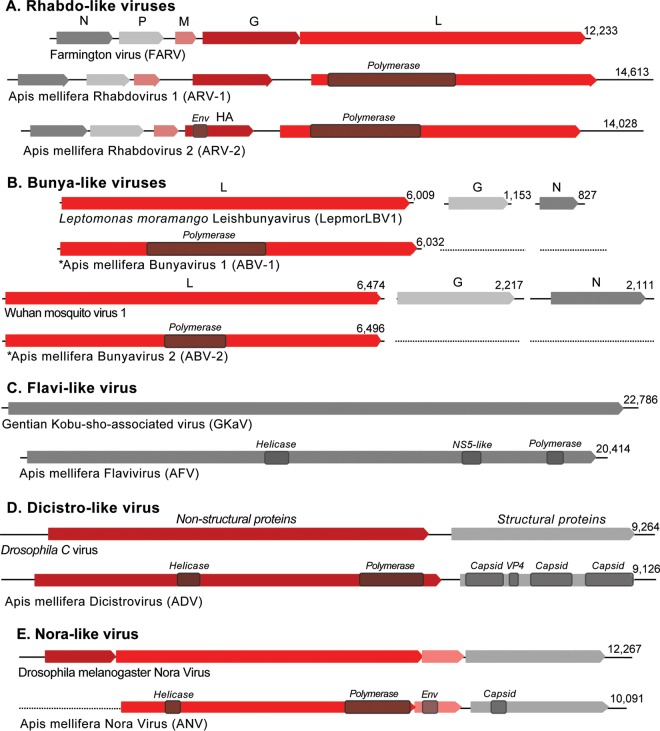
Genome structures of novel viruses. (A) Genome structures of rhabdo-like viruses, showing the genome size (nucleotides) and rhabdovirus open reading frames (N, P, M, G, and L/RdRp proteins) of ARV-1 (GenBank accession numbers KY354230 to KY354233) and ARV-2 (accession number KY354234) relative to the structure of the previously characterized Farmington virus. (B) Genome structure of bunya-like viruses, showing the identified L segment sizes (nucleotides) and the ORFs of ABV-1 (GenBank accession number KY354236) relative to LepmorLBV1 and of ABV-2 (accession number KY354237) relative to Wuhan mosquito virus 1. (C) Genome structure of a flavi-like virus, AFV (GenBank accession number KY354238), showing the genome size (nucleotides) and ORF relative to GKaV. (D) Genome structure of a dicistro-like virus, ADV (GenBank accession number KY354239), showing the genome size (nucleotides) and two ORFs of ADV relative to Drosophila C virus. (E) Genome structure of a Nora-like virus, ANV (GenBank accession number KY354240), showing the putative 5′-truncated genome size (nucleotides) and four ORFs relative to Drosophila Nora virus. (The NCBI protein sequence accession numbers are ARO50020 to ARO50052.)

**FIG 3:**
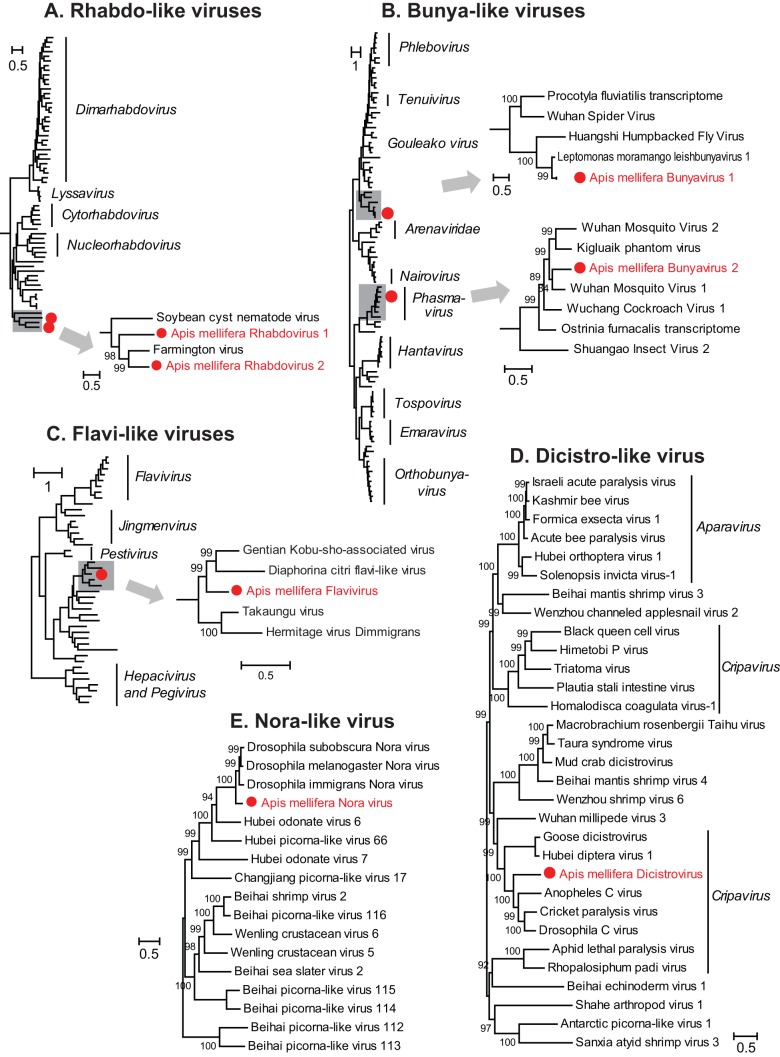
Evolutionary relationships of novel viruses. Shown are maximum likelihood phylogenies of the novel rhabdoviruses ARV-1 and ARV-2 (A), the novel bunyaviruses ABV-1 and ABV-2 (B), the novel flavivirus AFV (C), the novel dicistrovirus ADV (D), and the novel Nora virus ANV (E). (See Fig. S1 to S3 in the supplemental material for detailed trees for panels A to C.)

Next, we examined contigs that showed similarity to previously characterized virus sequences from positive-sense, negative-sense, and double-stranded RNA genomes available in GenBank. We found genomic evidence of seven previously undescribed viruses in the honey bee transcriptomes, including four negative-sense and three positive-sense RNA viruses (Tables 1 and 2 and Fig. 2 and 3). Of the negative-sense RNA viruses, two belong to the family Rhabdoviridae of the order Mononegavirales (Table 2 and Fig. 2A), and two belong to the family Bunyaviridae (Table 2 and Fig. 2B). The three positive-sense RNA viruses include one virus belonging to the family Flaviviridae, one related to Nora viruses (picorna-like) found in Drosophila (53), and one belonging to the family Dicistroviridae of the order Picornavirales, with homology to Drosophila C virus (DCV) (Table 2 and Fig. 2C to E). For the new viruses described here, individual abbreviations are derived from the host species (e.g., Apis mellifera), followed by the name or category of virus and number if more than 1 (e.g., rhabdovirus 1).

**TABLE 2:** Classifications, genome characteristics, and geographic locations of the novel viruses discovered in this study

Virus	Classification^*a*^			Characteristic		Geographic location(s)	Species from which the virus was isolated
	Genome type	Order	Family	Genome size (bp)	Closest relative (% RdRp amino acid identity)		
ARV-1	−ssRNA	Mononegavirales	Rhabdoviridae	14,613	Farmington virus (30)	The Netherlands, South Africa, Tonga	Apis mellifera, Varroa destructor
ARV-2	−ssRNA	Mononegavirales	Rhabdoviridae	14,028	Farmington virus (23)	South Africa, Tonga, The Netherlands	Apis mellifera, Varroa destructor
ABV-1	−ssRNA	Unclassified	Bunyaviridae	6,032^*b*^	Leishbunyavirus (56)	South Africa	Apis mellifera
ABV-2	−ssRNA	Unclassified	Bunyaviridae	6,496^*b*^	Wuhan mosquito virus 1 (42)	South Africa	Apis mellifera
AFV	+ssRNA	Unclassified	Flaviviridae	20,414	Gentian Kobu-sho-associated virus (20)	South Africa	Apis mellifera
ADV	+ssRNA	Picornavirales	Iflaviridae	9,126	Drosophila C virus (57)	The Netherlands	Apis mellifera
ANV-1	+ssRNA	Unclassified	Picorna-like	10,091^*c*^	Drosophila subobscura Nora virus (52)	South Africa	Apis mellifera

a −ssRNA, negative-sense ssRNA; +ssRNA, positive-sense ssRNA.

b Partial genome identified (L segment of bunyavirus).

c Partial genome identified; 5′ region incomplete.

### Novel negative-sense RNA viruses. (i) Rhabdo-like viruses.

**FIG 4:**
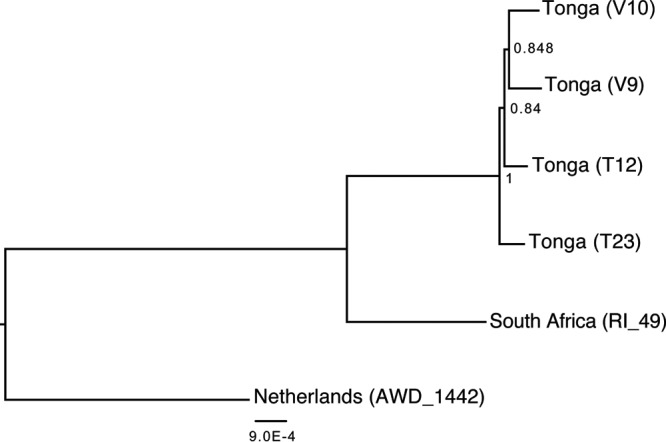
Apis mellifera rhabdovirus 1 variants. Shown is a maximum likelihood phylogenetic tree of the nucleotide alignment of ARV-1 variant genomes isolated from The Netherlands (GenBank accession number KY354230), South Africa (accession number KY354231), and Tonga (accession number KY354232).

Apis mellifera rhabdovirus 1 (ARV-1) has a 14,613-nt genome with a prototypic rhabdovirus structure (54) corresponding to the conserved gene order with five ORFs (Fig. 2A). The most conserved ORF encodes a 2,143-amino-acid (aa) protein containing the RNA-dependent RNA polymerase (RdRp) domain. ARV-1 RdRp was most similar to that of Farmington virus (FARV), a virus originally isolated from birds (55), with 30% amino acid identity (Table 2). The predicted G protein of ARV-1 was also most similar to that of FARV (18%); however, BLAST searches of the other ORFs showed no significant similarity to any known sequences. ARV-2 consists of a 14,029-nt genome with five predicted ORFs (Fig. 2A). The RdRp of ARV-2 was also related to that of FARV but with much lower sequence similarity (23% amino acid identity) (Table 2) than for ARV-1. Unlike the ARV-1 G protein, the ARV-2 G protein showed structural and sequence similarities to the hemagglutinin protein of Quaranfil virus of the Orthomyxoviridae family (22% identity), which suggests that this glycoprotein gene may be acquired through an inter-virus-family horizontal transfer event (33). Interestingly, both ARV-1 and ARV-2 show evidence of being widespread. ARV-1 was found in six colonies and in all three geographically diverse locations (The Netherlands, South Africa, and Tonga) (Tables 2 and 3), with a high abundance ranging from 50 to 500 transcripts per million (TPM) (Table 3). ARV-2 was moderately abundant in South Africa and Tonga (5 to 17 TPM) and was also detected in The Netherlands (1.8 TPM) (Tables 2 and 3). The ARV-1 and ARV-2 genomes from each location exhibit 98 to 99% nucleotide identity to each other (Fig. 4). The RdRp protein sequences of ARV-1 and ARV-2 formed a monophyletic group with that of FARV, which were distantly related to other members of the order Mononegavirales (Fig. 3A; see also Fig. S1 in the supplemental material).

**TABLE 3:** Abundances and prevalences of novel viruses in sampled colonies

Novel virus	Location	Sample	Abundance estimation (TPM)	Avg fold coverage
ARV-1	The Netherlands	NE_AWD_1442	47.75	235
	South Africa, RI	SA_RI_49	132.42	571
	Tonga, Vava'u	T_V9	186.4	705
		T_V10	55.3	263
	Tonga, Tongatapu	T_T12	546.98	3,023
		T_T23	348.21	2,232
ARV-2	South Africa, RI	SA_RI_49	8.73	62
	Tonga, Vava'u	T_V10	17.09	86
	Tonga, Tongatapu	T_T12	5.49	32
	The Netherlands	NE_AWD_1442^*a*^	1.79	5
ABV-1	South Africa, RI	SA_RI_A	65.51	398
		SA_RI_11	57.78	337
		SA_RI_49^*a*^	1.25	1
ABV-2	South Africa, RI	SA_RI_A	222.05	1,375
AFV	South Africa, RI	SA_RI_A	30.9	187
ADV	The Netherlands	NE_AWD_1151	1.71	13
ANV	South Africa, RI	SA_RI_11^*a*^	1.53	4

a Multiple were contigs formed (partial genome). The TPM reported is the average for all contigs.

### (ii) Bunya-like viruses.

We identified two bunya-like virus sequences, both of which were obtained from colonies from Robben Island (Fig. 2B). Members of the Bunyaviridae are negative-sense RNA viruses with three genome segments (L, M, and S), each containing a separate ORF (31). Due to a lack of sequence similarity to known sequences, for both viruses, we identified only the larger L segments encoding the RdRp domains. Apis mellifera bunyavirus 1 (ABV-1) was present in three colonies (Table 3). ABV-1 was most similar to a recently characterized class of bunyaviruses, the leishbunyaviruses, identified in the insect trypanosomatid parasite Leptomonas moramango (56). The RdRp protein of ABV-1 exhibits 56% amino acid identity to Leptomonas moramango leishbunyavirus 1 (LepmorLBV1) (Table 2). ABV-2 was present in one colony from Robben Island. The RdRp protein has 42% identity to Wuhan mosquito virus 1 (Table 2) (31). Phylogenetic analysis involving representative members from the Bunyaviridae placed ABV-1 in a clade with the leishbunyaviruses as a clade basal to other bunyaviruses found within invertebrates (31, 56) and placed ABV-2 in the cluster of Phasmavirus-like bunyaviruses (Fig. 3B and Fig. S2).

### Novel positive-sense RNA viruses. (i) Flavi-like virus.

Apis mellifera flavivirus (AFV) was identified in one colony from Robben Island. The 20,414-nt positive-sense RNA genome contains a single ORF of 6,615 aa (Fig. 2C). BLAST searches indicated that AFV has 20% amino acid identity to gentian Kobu-sho-associated virus (GKaV), a recently identified flavi-like virus originally thought to be a dsRNA virus (Table 2) (35, 57, 58). Similarly to GKaV and other newly identified flaviviruses, the 20.4-kb AFV-1 genome is longer than the typical length of previously characterized members of the Flaviviridae (35). The phylogeny based on the RdRp/NS5 domain of AFV and other members of the Flaviviridae placed AFV in a clade of other recently discovered flavi-like viruses with large genomes (35) (Fig. 3C; see also Fig. S3 in the supplemental material) and flavi-like virus segments identified in Drosophila species (Takaungu virus and Hermitage virus) (34).

### (ii) Dicistro-like virus.

We identified a novel dicistrovirus from one colony at the Amsterdam Water Dunes (Apis mellifera dicistrovirus [ADV]). The 9,126-nt genome contains two ORFs encoding the replication enzyme polypeptide and the capsid proteins, respectively, which is typical of dicistroviruses (Fig. 2D). The polypeptide containing the RdRp exhibited the highest genetic identity to Drosophila C virus (57% amino acid identity) (Table 2). Phylogenetic analysis placed ADV in the same clade as Cricket paralysis virus (CrPV), Drosophila C virus, and Anopheles C virus (Fig. 3D).

### (iii) Nora-like virus.

Twelve separate contigs were assembled from one colony from Robben Island, each of which showed similarity to Drosophila Nora virus after BLASTx analysis. To assemble a full-length genome, these contigs were ordered according to their most closely related virus, Drosophila pseudoobscura Nora virus, and gaps were filled by using reverse transcription-PCR (RT-PCR) and Sanger sequencing using primers spanning the neighboring contigs. The resulting Apis mellifera Nora virus (ANV) partial genome sequence is 10,091 nt long and covers the entire replicase, although it is missing the first ORF at the 5′ end of a typical Nora virus genome (Fig. 2E) (53). In the phylogenetic tree, the ANVs were closely related (52% to ∼54% amino acid identity) to Nora viruses isolated from different Drosophila species (Fig. 3E).

### Small RNA profiles of ARV-1 and ARV-2 in honey bees.

One way of confirming that a putative virus genuinely infects the host from which it is sampled is the presence of an antiviral immune response. In insects, likely candidates are the small RNA pathways that are utilized in viral defense (37, 40, 59). We therefore determined the presence of antiviral small RNAs in bees infected with our novel viruses. We focused on ARV-1 and ARV-2 because they are the first negative-sense RNA viruses described in honey bees and were present in colonies sampled from all three of our geographically diverse locations (Table 1).

We generated small RNA libraries from the abdomens of four A. mellifera samples: two from the Amsterdam Water Dunes and two from Robben Island. For each of these geographic locations, we used PCR to screen for individuals that were positive for ARV-1 and used one individual that tested positive for ARV-1 (Amsterdam Water Dunes positive [AWD^+^] and Robben Island positive [RI^+^]) and one individual that tested negative (Amsterdam Water Dunes negative [AWD^−^] and Robben Island negative [RI^−^]). The RI^+^ sample also tested positive for ARV-2. The four libraries were subjected to 50-bp single-end sequencing, resulting in between 10 million and 18 million reads per sample.

**FIG 5:**
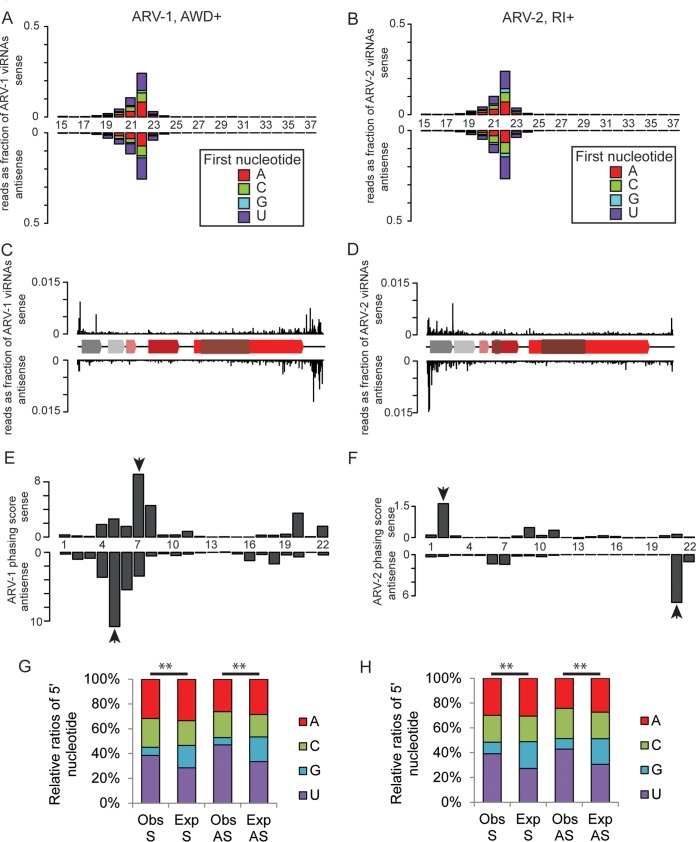
Small RNA analysis of ARV-1 and ARV-2 in honey bees. (A and B) Size distributions (15 to 37 nt) and 5′-nucleotide compositions of small RNAs arising from ARV-1 (A) and ARV-2 (B). The sample from which the small RNA library was produced is shown above each graph. Bars plotted above the *x* axis represent reads that map to the positive strand, and those plotted below represent those that map to the negative strand. Bars are colored according to the proportions of reads starting with A, C, G, and T. (C and D) Mapping of 20- to 23-nt-long viral RNAs (viRNAs) to the genomes of ARV-1 (C) and ARV-2 (D). The cartoon shows the domains of the viral genomes as shown in Fig. 2. (E and F) Phasing score over 8 phasing cycles for each position within a 22-nt phasing window. The top and bottom graphs show the phasing scores for the sense and antisense reads, respectively. A high score indicates that many small RNAs fall into that phase position (indicated with arrowheads). This analysis was performed by using the 21- to 23-nt-long reads from panels A and B. (G and H) Observed 5′ nucleotide (Obs) compared with that expected (Exp) from the base compositions of the viral genomes for ARV-1 (G) and ARV-2 (H). Sense (S) and antisense (AS) reads were compared by using a chi-squared test. **, *P* value of <0.01.

We first mapped the resulting small RNA reads to the Apis mellifera genome and then aligned the unmapped reads to the ARV-1 and ARV-2 genomes (Table 4). From this, we found highly abundant small RNAs mapping to ARV-1 and ARV-2 (Table 5). Such small RNAs could either be random degradation products of viral RNA or result from the antiviral immune response of honey bees. Random degradation products of negative-sense RNA viruses would show a mixed size distribution of predominantly negative-sense fragments spanning the entire viral genome. In contrast, our small RNA reads have a size distribution of 21 to 22 nt, occur equally in sense and antisense orientations, and map predominantly to the 5′ and 3′ ends of the ARV-1 and -2 genomes (Fig. 5A to D). These features are typical signatures of Dicer-produced antiviral RNAs, which occur when Dicer binds to a double-stranded RNA intermediate and cleaves the double-stranded RNA into viral siRNA (39).

**TABLE 4:** Small RNA samples and data generated

Sample	Data generated (no. of reads)	No. of reads mapped to the genome	% of reads mapped to the genome	No. of unmapped reads	No. of unmapped reads mapped to novel viruses	% of total reads mapped to novel viruses
AWD^+^	10,395,269	3,101,145^*a*^	30	7,294,124	80,473	1.1
AWD^−^	13,158,211	4,482,538^*a*^	34	8,675,673	25	0.0
RI^+^	17,633,773	4,945,358^*a*^	28	12,688,415	1,565,107	12.3
RI^−^	12,454,373	885,663^*a*^	7	11,568,710	198	0.0
M1	18,673,943	8,345,545^*b*^	45	10,328,398	379,229	2.6
M2	20,061,865	12,704,399^*b*^	63	7,357,466	71,471	0.5

a Apis mellifera genome.

b Varroa destructor genome.

**TABLE 5:** Number of small RNA reads mapped to ARV-1 and ARV-2

Sample	No. of small RNA reads mapped to^*a*^:	
	Apis mellifera rhabdovirus 1	Apis mellifera rhabdovirus 2
AWD^+^	80,473	NA
AWD^−^	25	NA
RI^+^	1,550,604	14,503
RI^−^	177	21
M1	202,052	177,177
M2	34,272	37,199

a NA, not applicable.

The 5′ and 3′ genome biases suggest that replication intermediates at the ends of the ARV-1 and ARV-2 genomes provide dsRNA termini for Dicer to bind. Dicer-produced antiviral RNAs occur at regularly spaced 21- to 23-nt intervals (phases) starting at the dsRNA termini and fading with increasing distance from the termini, with a characteristic 2- to 3-nt overhang (37). We looked for evidence of small RNAs occurring at regularly spaced intervals from the 5′ end of the ARV-1 and ARV-2 genomes using phasing analysis (60). We detected a strong phasing signature for ARV-1, 7 nt from the 5′ end, with a 2-nt overhang between the sense and antisense strands (Fig. 5E). We also detected a phasing signature for ARV-2, 2 nt from the 5′ end, with an offset of 3 nt between the sense and antisense strands (Fig. 5F). These data strongly indicate that Dicer is responsible for producing the 22-nt small RNAs.

Finally, the antiviral immune response is also mediated by RNA-binding proteins such as Argonaute proteins, which bind to small RNAs and induce the degradation of RNA sequences complementary to the small RNA (61). Argonaute proteins often show a 5′-nucleotide preference in small RNA molecules (61), so we looked for nucleotide bias at the 5′ end of our small RNA compared to the base composition of the viral genome. Both the sense and antisense small RNAs against ARV-1 and ARV-2 display a highly significant reduction in 5′-G and a highly significant increase in 5′-U as the 5′ nucleotides (*P* < 0.01 for both by a chi-squared test) (Fig. 5G and H).

Taken together, our data suggest that the ARV-1 and -2 small RNAs have been generated by Dicer acting on a double-stranded RNA replication intermediate and that the small RNAs are subsequently bound by Argonaute proteins, indicating that the bees have an active antiviral immune response against ARV-1 and ARV-2.

### Small RNA profiles of ARV-1 and ARV-2 in mites.

**FIG 6:**
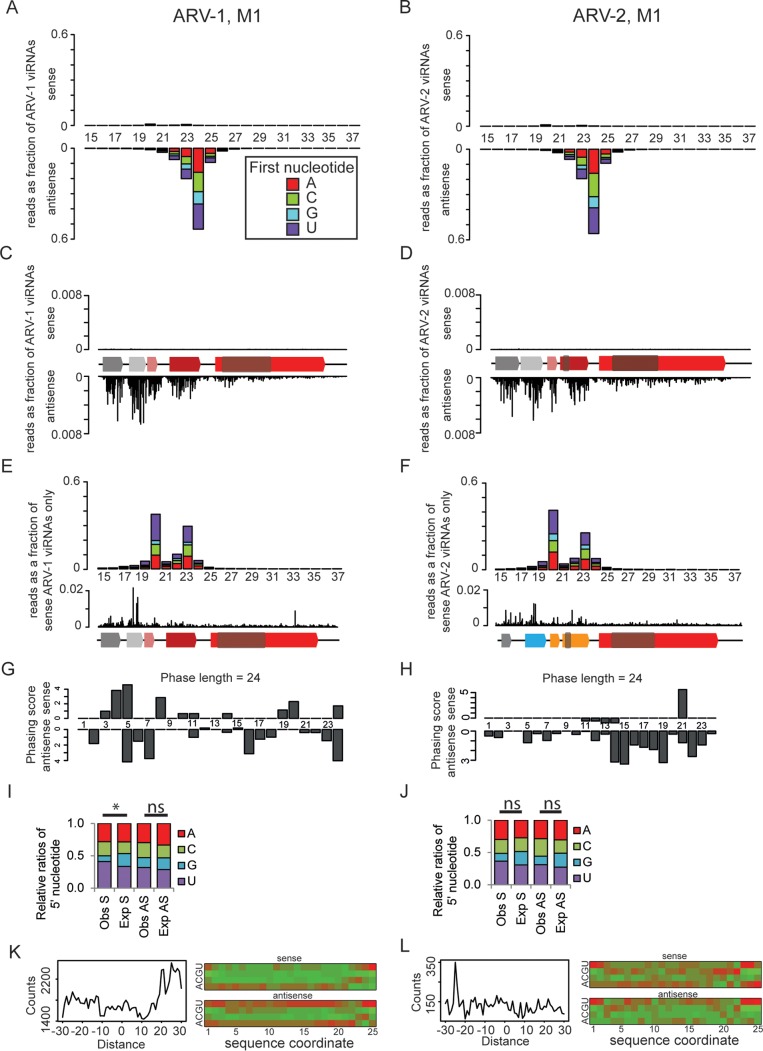
Small RNA analysis of ARV-1 and ARV-2 in Varroa. Left panels show ARV-1, and right panels show ARV-2. (A and B) Size distributions (15 to 37 nt) and 5′-nucleotide compositions of small RNAs in mite 1 (M1) arising from ARV-1 (A) and ARV-2 (B). Bars plotted above the *x* axis represent reads that map to the positive strand, and those plotted below represent those that map to the negative strand. Bars are colored according to the proportions of reads starting with A, C, G, and T. (C and D) Mapping of 23- to 25-nt-long viRNAs to the genomes of ARV-1 (C) and ARV-2 (D). The cartoon shows the domains of the viral genomes as shown in Fig. 2. (E and F) Size distributions and 5′-nucleotide compositions of the sense small RNAs from panels A and B, respectively, normalized to the number of sense reads present. Mapping of the 19- to 24-nt sense reads to the viral genomes is also shown. (G and H) Phasing scores over 8 phasing cycles for each position within a 24-nt phasing window for ARV-1 (G) and ARV-2 (H). The top and bottom graphs show the phasing scores for the sense and antisense reads, respectively. This analysis was performed by using the 24-nt-long reads only. (I and J) Observed 5′ nucleotides (Obs) compared with those expected (Exp) from the base compositions of the viral genomes of ARV-1 (I) and ARV-2 (J). Sense (S) and antisense (AS) reads were compared by using a chi-squared test. *, *P* value of <0.05; ns, not significant. (K and L) Distances between the 5′ ends of overlapping reads on opposite strands (left) and the base compositions of each nucleotide position (right) for the 23- to 25-nt-long reads of ARV-1 (K) and ARV-2 (L).

We next wanted to determine if ARV-1 and -2 are also found in mites feeding on infected bees. To this end, we generated small RNA libraries from two V. destructor mites collected from A. mellifera individuals from Robben Island and performed Illumina 50-bp single-end sequencing, resulting in 18 million to 20 million reads per sample (Table 4). We found small RNA reads mapping to ARV-1 and ARV-2 in both mite samples (Table 5 and Fig. 6).

The small RNA reads present in the mites show markedly different characteristics compared to the small RNA patterns in the honey bee samples. In mites, the antisense reads vastly outnumber the sense reads, span the length of the genome, and have a distinct length distribution centered at 24 nt. Furthermore, the antisense reads correspond to the first four ORFs encoding the ARV-1 and ARV-2 proteins (Fig. 6A to D). The sense reads, while much less abundant, show a broader size range than the antisense reads, with peaks at 20 and 23 nt, and do not localize specifically to the ORFs (Fig. 6E and F and data not shown). Phasing analysis from the 5′ end of the viral RNA did not show any evidence of phased RNAs of any size for either sense or antisense reads (Fig. 6G and H). Furthermore, there was only a weak 5′-nucleotide bias against 5′-G and toward 5′-U compared to the base composition of the viral genome in sense reads for ARV-1 (*P* < 0.05 by a chi-squared test), no 5′-nucleotide bias for antisense reads for ARV-1 (not significant [NS] by a chi-squared test), and no 5′-nucleotide bias for sense or antisense reads mapping to ARV-2 (NS by a chi-squared test) (Fig. 6I and J). The lack of phasing, weak 5′-nucleotide bias, and small quantity of sense reads suggest that the Dicer and Argonaute antiviral pathways do not act on replicating ARV-1 and ARV-2 dsRNAs in mites.

We also tested the 23- to 25-nt-long reads for signatures of the piwi-interacting RNA (piRNA) ping-pong amplification pathway, as this pathway has been implicated in viral defense in Aedes aegypti (62, 63). These signatures include a bias for uridine at the 5′ end and an adenine at position 10 on the complementary piRNA as well as a 10-nucleotide distance between the 5′ ends of overlapping sense and antisense reads (64). We generated a heat map showing the relative enrichment of each nucleotide at each position in the 23- to 25-nt-long small RNAs. Although we saw some evidence of U enrichment at position 1, there was no evidence to suggest an enrichment of A at position 10. We also plotted the distance between the 5′ ends of overlapping 23- to 25-nt-long RNAs that map to ARV-1 and ARV-2 but found no evidence for a peak at 10 nt. Thus, we could not detect any evidence of a “ping-pong” signature in the reads originating from either ARV-1 or ARV-2 in either mite sample (Fig. 6K and L and data not shown). Taken together, these data could mean that the small RNAs from ARV-1 and ARV-2 detected in mites are simply the products of random degradation, which is most likely the case for the sense small RNAs. However, in the antisense reads, the strong bias for 24-nt RNAs argues against random degradation.

The small RNA profiles in mites do not mimic those in honey bees, indicating that the mite reads do not come from ingested honey bee material. Thus, the high abundance of reads in the mites (Table 4) and the presence of sense reads (showing that a sense genome has been produced) suggest that both mites and honey bees are bona fide hosts of ARV-1 and ARV-2.

## DISCUSSION

We describe a diverse set of new viruses in Varroa-resistant or Varroa-free honey bee populations from three locations in Europe, Africa, and the Pacific. We present genomic evidence of seven new RNA viruses, including three novel positive-sense and four novel negative-sense viruses. Our study therefore increases the number of known honey bee viruses from 24 to 31. ARV-1 and -2 were found in three geographically distinct populations. Using small RNA sequencing, we show that honey bees exhibit classic Dicer-mediated siRNA profiles, suggesting an active bee immune response. We also report the first analysis of small RNAs in Varroa destructor mites and show that ARV-1 and -2 are present in these mites, although the small RNA profile is distinctly different from that for honey bees.

To our knowledge, this is the first identification of negative-sense viruses in honey bees. Three of the four novel negative-sense viruses (ARV-1, ARV-2, and ABV-2) are related to viruses known to be present in insects (65). Our findings are thus consistent with data from recent studies describing the wide distribution of negative-sense viruses in arthropod hosts (31, 32, 66). Indeed, two negative-sense viruses were also recently found in the wild solitary bee Osmia cornuta, including one virus from the order Mononegavirales and one from the family Bunyaviridae, indicating that viral diversity in other hymenopteran species extends to negative-sense viruses (67). One of the novel negative-sense viruses from the Bunyaviridae identified in our South African population, ABV-1, was closely related to a recently isolated protist-infecting virus, the leishbunyavirus LepmorLBV1 (56). LepmorLBV1 was isolated from the insect trypanosomatid parasite Leptomonas moramango, a parasite of Brachycera flies (68). Interestingly, the three colonies from Robben Island that contained ABV-1 contigs were also positive for the honey bee trypanosome Lotmaria passim (52). We therefore cannot exclude that ABV-1 is a virus of protists that infect bees.

The small RNA patterns of the rhabdoviruses in honey bees show classical Dicer-mediated degradation profiles, providing strong evidence that ARV-1 and -2 enter the cells of bees and begin to replicate. During viral replication, a double-stranded RNA replication intermediate is formed, which can be recognized by the RNAi machinery and chopped by Dicer. The resulting small RNAs become part of an antiviral immune response (59). This strongly suggests that these novel negative-sense viruses are bona fide viruses capable of replication in honey bees.

In contrast, in Varroa, the strong bias for 24-nt antisense small RNAs and the lack of phasing suggest that replicating dsRNA is not a template for Dicer. Internal secondary structures within a negative-sense virus genome can provide dsRNA templates for Dicer (69, 70). An alternative Dicer-mediated antiviral response seems to be the most parsimonious explanation for the small RNA profile found for the mites. Interestingly, however, the predominant size of 24-nt antisense reads is larger than expected for canonical Dicer products. It is unclear whether Dicer in Varroa destructor produces longer-than-usual RNA fragments or if these 24-nt RNAs are generated by a different viral degradation pathway. Other mites, such as Tetranychus and Metaseiulus mites, contain the components of the RNAi machinery, with considerable variation in gene copy numbers for Dicer and Argonaute proteins (71). An important step toward understanding the Varroa RNA interference pathway will be determining if the Varroa genome contains similar variations in key RNAi-mediating genes. It is intriguing that the 24-nt antisense reads correspond to the ORFs of ARV-1 and -2. Similar “hot spots” have been observed previously, but their functional relevance is unclear (72).

Our study substantially expands the taxonomic diversity of honey bee viruses. Until now, most characterized honey bee viruses were restricted to the order Picornavirales (18, 19). The invertebrate-specific Dicistroviridae and Iflaviridae classes are evidently well adapted to parasitizing insects, and many viruses in these groups show extremely broad host ranges (73–76), which may facilitate spread, allow viruses to replicate more readily in multiple hosts, and thus allow viruses to become more prevalent and easily detected. Positive-sense RNA viruses are also more abundant in eukaryotes generally (77), which likely contributes to the frequency with which they are detected in honey bees. Indeed, we found three novel positive-sense RNA viruses. Two of these viruses, ANV and ADV, show relatively close evolutionary relationships with similar viruses from Drosophila. Interestingly, early serological characterizations of DCV and the related CrPV included honey bee samples for cross-reactivity to CrPV and DCV sera and identified a honey bee variant (73, 78, 79). The third positive-sense virus identified here, AFV, follows from the recent identification of larger flavivirus genomes (35), suggesting that arthropods could harbor a variety of viruses that will further illuminate the evolutionary origins of common viral categories (31, 35).

Until now, viral surveys in honey bees predominantly focused on PCR-based approaches, which were heavily dependent on existing virus diversity (2, 3, 16, 25, 27) or on infectivity tests in honey bees, where viruses were identified based on their ability to multiply after injection into adult bees or pupae (18). This approach would necessarily exclude viruses that require different preparation methods or that are not amenable to crude extraction. Importantly, our use of metagenomic techniques was crucial in revealing a more complete bee virome, as many highly divergent viruses can be detected only at the sequence level (43, 46). Our study was also aided by the recent explosion in novel virus genomes (31–33, 35, 45), which provides a more comprehensive database for BLAST searches. As this database continues to expand, it is likely that more new viruses in a range of host species will be isolated. Finally, our detection of novel viruses may in part reflect our focus on bee colonies that are resistant to, or free of, Varroa, as the rapid spread of some viruses in the context of Varroa, most notably virulent variants of DWV, may have resulted in a general reduction in virus diversity. Clearly, the discovery of the new viruses here suggests that the bee virome will continue to expand following more extensive metagenomic surveys in diverse geographic regions.

## MATERIALS AND METHODS

### Sample collection.

Honey bee colonies and mites from Europe, Africa, and the Pacific were sampled in 2013 to 2015. Seven A. mellifera capensis colonies from Robben Island and five colonies from mainland South Africa were sampled in March 2013. Varroa mites were first identified in South Africa in 1997, and after initial deleterious effects, A. mellifera capensis colonies exhibited natural mite resistance after 3 to 5 years (49). On Robben Island, Varroa mites were first detected 2 years prior to sampling (M. Allsopp, personal communication). In July 2014, 10 colonies of A. mellifera bees from an apiary at the Amsterdam Water Dunes, The Netherlands, where Varroa has been present since the 1980s and where natural selection for Varroa-resistant colonies has been ongoing since 2008, were sampled (48). In October 2015, feral and managed A. mellifera colonies from islands in the Kingdom of Tonga were sampled. Nine colonies from Vava'u island, where Varroa destructor was introduced in 2006, were sampled. Honey bees on Vava'u exhibit a natural tolerance that has enabled the survival of colonies in the decade following the introduction of the mites. Four colonies from Tongatapu island, where Varroa is not yet present, were sampled. In Tonga, 10 adult worker honey bee thoraxes per colony were crushed individually in 500 μl of RNAlater (Qiagen) and transported at room temperature prior to storage at −80°C until processing. In Africa and The Netherlands, a minimum of 10 adult worker honey bees were sampled per colony, frozen immediately on dry ice, and stored at −80°C until processing.

### Sample processing.

Thorax and abdomen tissues of five adult bees from each colony (thorax only from Tonga) were homogenized in TRIzol reagent (Thermo Fisher), and total RNA was extracted according to the manufacturer's protocol. RNA from each individual was diluted to 200 ng/μl, and 2.65 ng RNA from each of the five individuals from each colony was pooled prior to DNase treatment (Ambion), followed by column purification (RNeasy minikit; Qiagen). Total RNA was transported to the Australian Genome Research Facility (AGRF) on dry ice. Sample RNA integrity was confirmed by using a Bioanalyzer (Agilent). RNA was subjected to a ribosome depletion step (Ribo-Zero-Gold Human/Mouse/Rat) prior to the preparation of Illumina TruSeq Stranded Total RNA paired-end libraries according to the manufacturer's instructions. Libraries were run on an Illumina HiSeq2000 100-bp paired-end sequencing system for a total data yield of 4 to 9 Gb per sample.

### Sequence read assembly and virus discovery.

Sequencing reads were assembled *de novo* by using Trinity (80). The resulting contigs were compared to reference protein sequences of all previously characterized viruses downloaded from GenBank by using BLASTx (with an E value of 1E−5 to maximize sensitivity while minimizing false-positive results [35]). The resulting virus-like contigs were then compared to a nonredundant database using BLAST to remove nonviral hits, such as host contigs with similarity to viral sequences. We also removed any contigs with high similarity to plant viruses, which were more likely to be derived from food sources (although there is the possibility that these viruses could be replicating in bees [see reference 81]).

Virus sequences were aligned to sequences present in the current NCBI databases of homologous viral proteins by using MAFFT (82). Alignments were viewed, manually trimmed to remove large gaps and nonconserved regions, and further trimmed with TrimAL to remove ambiguously aligned regions (83). Maximum likelihood phylogenetic trees for each data set were inferred by using PhyML (84) in parallel mode, using Message Passaging Interface (MPI) with 12 threads and 12 random starting trees. We used a best-fit model of amino acid substitution determined by using ProtTest (85), a Subtree Pruning and Regrafting (SPR) branch-swapping algorithm, and an approximate likelihood ratio test (aLRT) with the Shimodaira-Hasegawa-like procedure to assess branch support.

### Validation of novel viruses.

We used PCR and sequencing to confirm the presence of each novel virus, designing primers based on the contigs assembled from next-generation sequencing. One virus, ANV, produced fragmented contigs spanning an incomplete genome. Each contig was ordered based on translated homology to the most closely related virus, Drosophila pseudoobscura Nora virus, and primers spanning contig breaks were designed to confirm the correct genome order and to sequence any unassembled regions (data not shown). We also confirmed the arrangement of the ARV-2 G and L proteins and the putative inter-virus-family horizontal transfer event, using PCR for amplification across the two ORFs and Sanger sequencing for confirmation.

We used Bowtie2 (86) to map reads to each of the novel virus genomes, Samtools to determine the sequencing depth and coverage (87), and the RSEM program implemented in Trinity to estimate the abundance of virus transcripts per million (TPM) (Table 3) (51). ORFs (Fig. 1) were annotated based on predicted amino acid sequences that were more than 200 nucleotides long as well as conserved positions in the genome compared to the most closely related viruses. Conserved domains were identified by using NCBI CDD BLAST searches (88).

### Small RNA library preparation and sequencing.

One microgram of total RNA (prepared as described above) from individual bees and Varroa mites was used to generate a small RNA library by using a TrueSeq small RNA kit (Illumina) according to the manufacturer's protocol. Samples were barcoded appropriately for pooling on an Illumina HiSeq2000 instrument, with 50-bp single-end sequencing.

### Small RNA analysis.

Small RNA reads were quality checked, trimmed to remove the TruSeq adapter, and then mapped by using CLC Genomics Workbench (Qiagen). The reads were first mapped to the A. mellifera genome, allowing for up to two mismatches (length fraction of 1 and similarity fraction of 0.9) due to the divergence between honey bee strains and the reference genome. The unmapped reads were subsequently mapped to ARV-1 and ARV-2 with the same stringency settings. The Amsterdam Water Dunes and Robben Island samples were mapped to the consensus viral genome generated by RNA sequencing (see above) from their geographic location. For further small RNA analysis (nucleotide size, genome position, base composition, and 5′ read distance), the mapped reads were exported as BAM files, indexed by using Samtools (87), and then analyzed in RStudio using viRome (89) and custom scripts.

### Phasing analysis of small RNAs.

To determine whether the small RNAs produced from the viral genomes showed evidence of Dicer phasing, we adapted an algorithm designed for detecting phased RNAs in plants (60). The logic behind this analysis is that if an RNA molecule is being cut every 22 nucleotides, there should be a relative accumulation of small RNA reads every 22 nucleotides. Thus, we summed the number of reads every *x* + 22 nucleotides along the sequence (*P_i_*) (where the phase cycle position [*x*] equals 1 to 22) using custom R scripts and then divided this value by the number of remaining out-of-phase reads (*U*). Phasing scores were calculated for each phase cycle position in the 22-nt window over 8 cycles, as the signal is expected to degrade over time due to imprecision in the cut length. The relative accumulation of reads at a particular phase cycle position will give a high phasing score. For the honey bee data, we specifically looked for a phase cycle of 22, since this was the predominant size of the small RNAs. For the Varroa data, we analyzed four different phase cycle lengths (20, 22, 23, or 24 nucleotides) using the following equation:

(1) $$\text{phasing score}=\ln\left[{\left(1+10\frac{\Sigma_{i\;=\;1}^{8}\;P_{i}}{1\;+\;\Sigma U}\right)}^{n\;-\;2}\right]$$

where *i* is the phasing cycle, *P_i_* is the number of small RNA reads at a given phase cycle position, *U* is the number of small RNA reads within the phase cycle not at the phase cycle position (out of phase), and *n* is the number of phase cycle positions occupied by at least one small RNA within the 8 cycles.

### Accession number(s).

All virus genome sequences generated in this study have been deposited in the GenBank database under accession numbers KY354230 to KY354234 and KY354236 to KY354244. Annotated NCBI protein sequence accession numbers are ARO50020 to ARO50067. Raw sequence data have been deposited in the Sequence Read Archive (SRA) (accession number SRP095071) (BioProject accession number PRJNA357165 and BioSample accession numbers SAMN06140203 to SAMN06140219).

## Supplementary Material

Supplemental material
