# Supplementary material for: A Diverse Range of Novel RNA Viruses in Geographically Distinct Honey Bee Populations
Source: J Virol. 2017 Jul 27;91(16):e00158-17. doi: 10.1128/JVI.00158-17 (PMC5533899; doi:10.1128/JVI.00158-17)
Supplement: Supplemental material [file supp_91_16_e00158-17__index.html]

Supplemental material 

# A Diverse Range of Novel RNA Viruses in Geographically Distinct Honey Bee Populations

## Supplemental material

- Supplemental file 1 -

  Fig. S1 (Rhabdo-like viruses.)

  Fig. S2 (Bunya-like viruses.)

  Fig. S3 (Flavi-like viruses.)

  PDF, 725K
