# Supplementary material for: A Diverse Range of Novel RNA Viruses in Geographically Distinct Honey Bee Populations
Source: J Virol. 2017 Jul 27;91(16):e00158-17. doi: 10.1128/JVI.00158-17 (PMC5533899; doi:10.1128/JVI.00158-17)
Supplement: Supplemental material [file JVI.00158-17_zjv999182764s1.pdf]

SUPPLEMENTAL MATERIAL

Figure S1. Rhabdo-like viruses. Maximum likelihood phylogenetic tree of RdRp protein alignment of Rhabdoviruses. New Apis mellifera Rhabdoviruses 1 and 2 are indicated by red dots.

Figure S2. Bunya-like viruses. Maximum likelihood phylogenetic tree of RdRp protein alignment of Bunyaviruses. New Apis mellifera Bunyaviruses 1 and 2 are indicated by red dots.

Figure S3. Flavi-like viruses. Maximum likelihood phylogenetic tree of RdRp protein alignment of Flaviviruses. New Apis mellifera Flavivirus 1 is indicated by a red dot.

Figure S1

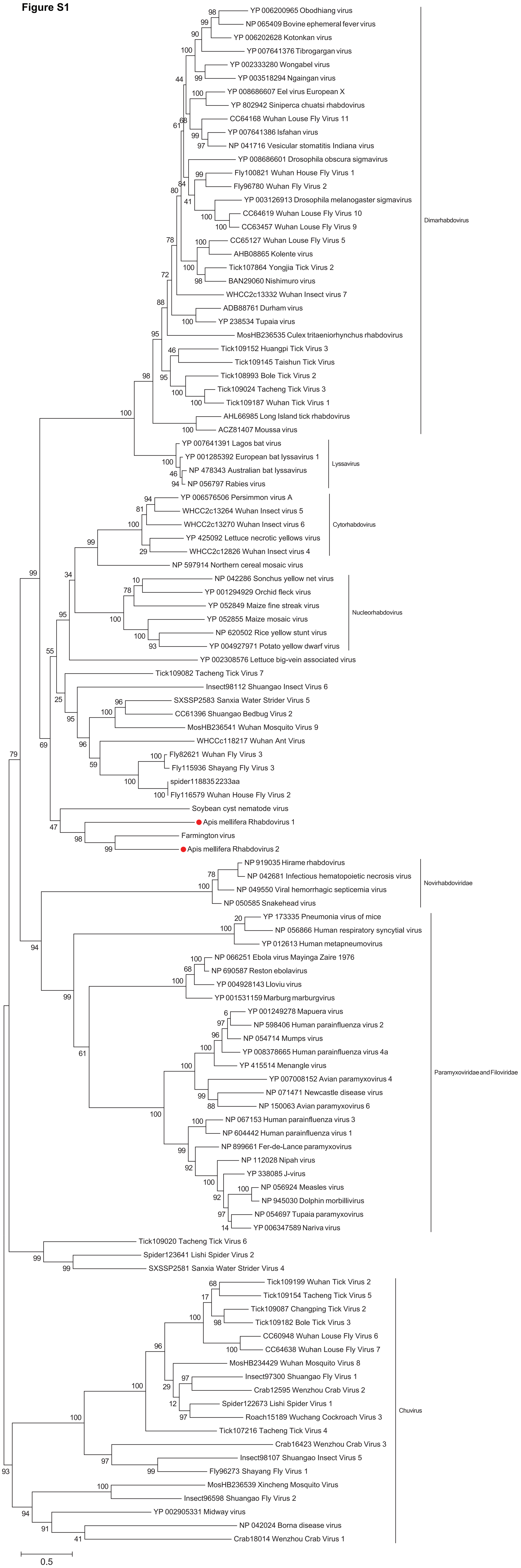

YP 006200965 Obodhiang virus
NP 065409 Bovine ephemeral fever virus
YP 006202628 Kotonkan virus
YP 007641376 Tibrogargan virus
YP 002333280 Wongabel virus
YP 003518294 Ngaingan virus
YP 008686607 Eel virus European X
YP 802942 Siniperca chuatsi rhabdovirus
CC64168 Wuhan Louse Fly Virus 11
YP 007641386 Isfahan virus
NP 041716 Vesicular stomatitis Indiana virus
YP 008686601 Drosophila obscura sigmavirus
Fly100821 Wuhan House Fly Virus 1
Fly96780 Wuhan Fly Virus 2
YP 003126913 Drosophila melanogaster sigmavirus
CC64619 Wuhan Louse Fly Virus 10
CC63457 Wuhan Louse Fly Virus 9
CC65127 Wuhan Louse Fly Virus 5
AHB08865 Kolente virus
Tick107864 Yongjia Tick Virus 2
BAN29060 Nishimuro virus
WHCC2c13332 Wuhan Insect virus 7
ADB88761 Durham virus
YP 238534 Tupaia virus
MosHB236535 Culex tritaeniorhynchus rhabdovirus
Tick109152 Huangpi Tick Virus 3
Tick109145 Taishun Tick Virus
Tick108993 Bole Tick Virus 2
Tick109024 Tacheng Tick Virus 3
Tick109187 Wuhan Tick Virus 1
AHL66985 Long Island tick rhabdovirus
ACZ81407 Moussa virus
Dimarhabdovirus
YP 007641391 Lagos bat virus
YP 001285392 European bat lyssavirus 1
NP 478343 Australian bat lyssavirus
NP 056797 Rabies virus
Lyssavirus
YP 006576506 Persimmon virus A
WHCC2c13264 Wuhan Insect virus 5
WHCC2c13270 Wuhan Insect virus 6
YP 425092 Lettuce necrotic yellows virus
WHCC2c12826 Wuhan Insect virus 4
Cytorhabdovirus
NP 597914 Northern cereal mosaic virus
NP 042286 Sonchus yellow net virus
YP 001294929 Orchid fleck virus
YP 052849 Maize fine streak virus
YP 052855 Maize mosaic virus
NP 620502 Rice yellow stunt virus
YP 004927971 Potato yellow dwarf virus
Nucleorhabdovirus
YP 002308576 Lettuce big-vein associated virus
Tick109082 Tacheng Tick Virus 7
Insect98112 Shuangao Insect Virus 6
SXSSP2583 Sanxia Water Strider Virus 5
CC61396 Shuangao Bedbug Virus 2
MosHB236541 Wuhan Mosquito Virus 9
WHCCc118217 Wuhan Ant Virus
Fly82621 Wuhan Fly Virus 3
Fly115936 Shayang Fly Virus 3
spider118835 2233aa
Fly116579 Wuhan House Fly Virus 2
Soybean cyst nematode virus
Apis mellifera Rhabdovirus 1
Farmington virus
Apis mellifera Rhabdovirus 2
NP 919035 Hirame rhabdovirus
NP 042681 Infectious hematopoietic necrosis virus
NP 049550 Viral hemorrhagic septicemia virus
NP 050585 Snakehead virus
Novirhabdoviridae
YP 173335 Pneumonia virus of mice
NP 056866 Human respiratory syncytial virus
YP 012613 Human metapneumovirus
NP 066251 Ebola virus Mayinga Zaire 1976
NP 690587 Reston ebolavirus
YP 004928143 Lloviu virus
YP 001531159 Marburg marburgvirus
YP 001249278 Mapuera virus
NP 598406 Human parainfluenza virus 2
NP 054714 Mumps virus
YP 008378665 Human parainfluenza virus 4a
YP 415514 Menangle virus
YP 007008152 Avian paramyxovirus 4
NP 071471 Newcastle disease virus
NP 150063 Avian paramyxovirus 6
NP 067153 Human parainfluenza virus 3
NP 604442 Human parainfluenza virus 1
NP 899661 Fer-de-Lance paramyxovirus
NP 112028 Nipah virus
YP 338085 J-virus
NP 056924 Measles virus
NP 945030 Dolphin morbillivirus
NP 054697 Tupaia paramyxovirus
YP 006347589 Nariva virus
Paramyxoviridae and Filoviridae
Tick109020 Tacheng Tick Virus 6
Spider123641 Lishi Spider Virus 2
SXSSP2581 Sanxia Water Strider Virus 4
Tick109199 Wuhan Tick Virus 2
Tick109154 Tacheng Tick Virus 5
Tick109087 Changping Tick Virus 2
Tick109182 Bole Tick Virus 3
CC60948 Wuhan Louse Fly Virus 6
CC64638 Wuhan Louse Fly Virus 7
MosHB234429 Wuhan Mosquito Virus 8
Insect97300 Shuangao Fly Virus 1
Crab12595 Wenzhou Crab Virus 2
Spider122673 Lishi Spider Virus 1
Roach15189 Wuchang Cockroach Virus 3
Tick107216 Tacheng Tick Virus 4
Chuvirus
Crab16423 Wenzhou Crab Virus 3
Insect98107 Shuangao Insect Virus 5
Fly96273 Shayang Fly Virus 1
MosHB236539 Xincheng Mosquito Virus
Insect96598 Shuangao Fly Virus 2
YP 002905331 Midway virus
NP 042024 Borna disease virus
Crab18014 Wenzhou Crab Virus 1
0.5

Figure S2

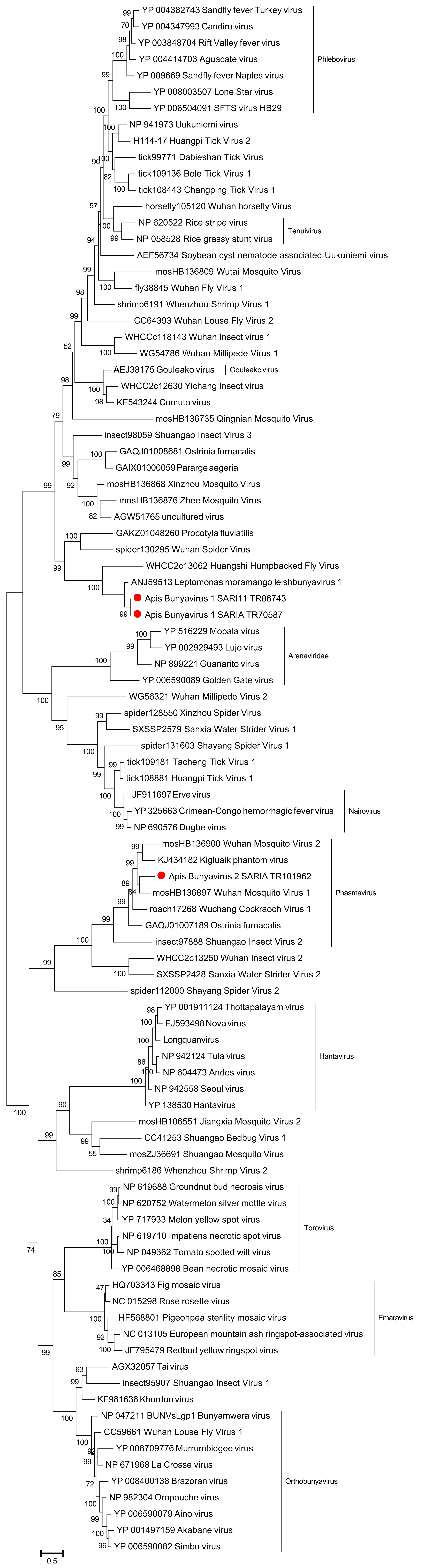
YP 004382743 Sandfly fever Turkey virus
YP 004347993 Candiru virus
YP 003848704 Rift Valley fever virus
YP 004414703 Aguacate virus
YP 089669 Sandfly fever Naples virus
YP 008003507 Lone Star virus
YP 006504091 SFTS virus HB29
Phlebovirus
NP 941973 Uukuniemi virus
H114-17 Huangpi Tick Virus 2
tick99771 Dabieshan Tick Virus
tick109136 Bole Tick Virus 1
tick108443 Changping Tick Virus 1
horsefly105120 Wuhan horsefly Virus
NP 620522 Rice stripe virus
NP 058528 Rice grassy stunt virus
Tenuivirus
AEF56734 Soybean cyst nematode associated Uukuniemi virus
mosHB136809 Wutai Mosquito Virus
fly38845 Wuhan Fly Virus 1
shrimp6191 Whenzhou Shrimp Virus 1
CC64393 Wuhan Louse Fly Virus 2
WHCCc118143 Wuhan Insect virus 1
WG54786 Wuhan Millipede Virus 1
AEJ38175 Gouleako virus
Gouleako virus
WHCC2c12630 Yichang Insect virus
KF543244 Cumuto virus
mosHB136735 Qingnian Mosquito Virus
insect98059 Shuangao Insect Virus 3
GAQJ01008681 Ostrinia furnacalis
GAIX01000059 Pararge aegeria
mosHB136868 Xinzhou Mosquito Virus
mosHB136876 Zhee Mosquito Virus
AGW51765 uncultured virus
GAKZ01048260 Procotyla fluviatilis
spider130295 Wuhan Spider Virus
WHCC2c13062 Huangshi Humpbacked Fly Virus
ANJ59513 Leptomonas moramango leishbunyavirus 1
Apis Bunyavirus 1 SARI11 TR86743
Apis Bunyavirus 1 SARIA TR70587
YP 516229 Mobala virus
YP 002929493 Lujo virus
NP 899221 Guanarito virus
YP 006590089 Golden Gate virus
Arenaviridae
WG56321 Wuhan Millipede Virus 2
spider128550 Xinzhou Spider Virus
SXSSP2579 Sanxia Water Strider Virus 1
spider131603 Shayang Spider Virus 1
tick109181 Tacheng Tick Virus 1
tick108881 Huangpi Tick Virus 1
JF911697 Erve virus
YP 325663 Crimean-Congo hemorrhagic fever virus
NP 690576 Dugbe virus
Nairovirus
mosHB136900 Wuhan Mosquito Virus 2
KJ434182 Kigluaik phantom virus
Apis Bunyavirus 2 SARIA TR101962
mosHB136897 Wuhan Mosquito Virus 1
roach17268 Wuchang Cockraoch Virus 1
GAQJ01007189 Ostrinia furnacalis
insect97888 Shuangao Insect Virus 2
Phasmavirus
WHCC2c13250 Wuhan Insect virus 2
SXSSP2428 Sanxia Water Strider Virus 2
spider112000 Shayang Spider Virus 2
YP 001911124 Thottapalayam virus
FJ593498 Nova virus
Longquanvirus
NP 942124 Tula virus
NP 604473 Andes virus
NP 942558 Seoul virus
YP 138530 Hantavirus
Hantavirus
mosHB106551 Jiangxia Mosquito Virus 2
CC41253 Shuangao Bedbug Virus 1
mosZJ36691 Shuangao Mosquito Virus
shrimp6186 Whenzhou Shrimp Virus 2
NP 619688 Groundnut bud necrosis virus
NP 620752 Watermelon silver mottle virus
YP 717933 Melon yellow spot virus
NP 619710 Impatiens necrotic spot virus
NP 049362 Tomato spotted wilt virus
YP 006468898 Bean necrotic mosaic virus
Torovirus
HQ703343 Fig mosaic virus
NC 015298 Rose rosette virus
HF568801 Pigeonpea sterility mosaic virus
NC 013105 European mountain ash ringspot-associated virus
JF795479 Redbud yellow ringspot virus
Emaravirus
AGX32057 Tai virus
insect95907 Shuangao Insect Virus 1
KF981636 Khurdun virus
NP 047211 BUNVsLgp1 Bunyamwera virus
CC59661 Wuhan Louse Fly Virus 1
YP 008709776 Murrumbidgee virus
NP 671968 La Crosse virus
YP 008400138 Brazoran virus
NP 982304 Oropouche virus
YP 006590079 Aino virus
YP 001497159 Akabane virus
YP 006590082 Simbu virus
Orthobunyavirus
0.5

Figure S3

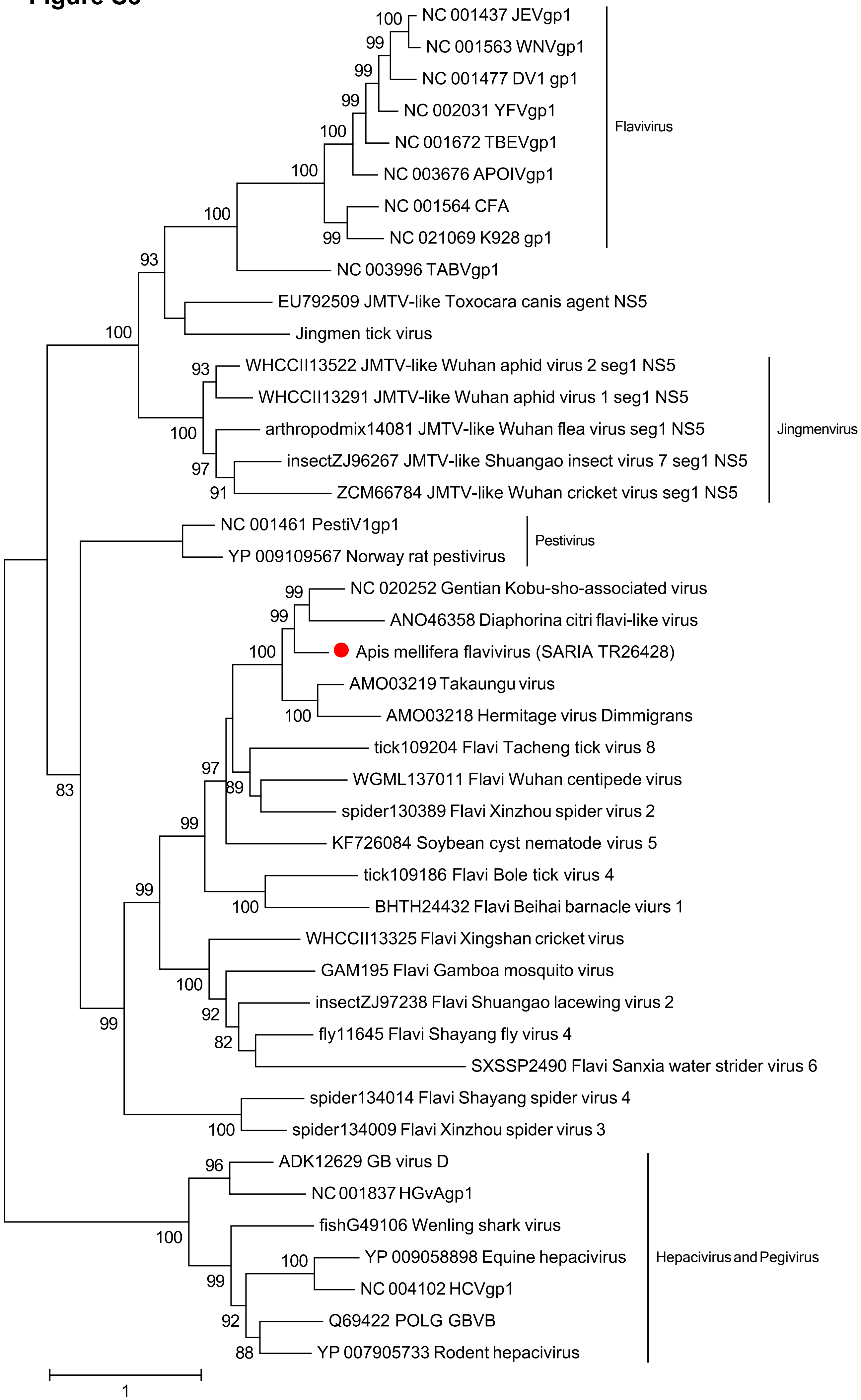
NC 001437 JEVgp1
NC 001563 WNVgp1
NC 001477 DV1 gp1
NC 002031 YFVgp1
NC 001672 TBEVgp1
NC 003676 APOIVgp1
NC 001564 CFA
NC 021069 K928 gp1
NC 003996 TABVgp1
EU792509 JMTV-like Toxocara canis agent NS5
Jingmen tick virus
WHCCII13522 JMTV-like Wuhan aphid virus 2 seg1 NS5
WHCCII13291 JMTV-like Wuhan aphid virus 1 seg1 NS5
arthropodmix14081 JMTV-like Wuhan flea virus seg1 NS5
insectZJ96267 JMTV-like Shuangao insect virus 7 seg1 NS5
ZCM66784 JMTV-like Wuhan cricket virus seg1 NS5
NC 001461 PestiV1gp1
YP 009109567 Norway rat pestivirus
NC 020252 Gentian Kobu-sho-associated virus
ANO46358 Diaphorina citri flavi-like virus
Apis mellifera flavivirus (SARIA TR26428)
AMO03219 Takaungu virus
AMO03218 Hermitage virus Dimmigrans
tick109204 Flavi Tacheng tick virus 8
WGML137011 Flavi Wuhan centipede virus
spider130389 Flavi Xinzhou spider virus 2
KF726084 Soybean cyst nematode virus 5
tick109186 Flavi Bole tick virus 4
BHTH24432 Flavi Beihai barnacle viurs 1
WHCCII13325 Flavi Xingshan cricket virus
GAM195 Flavi Gamboa mosquito virus
insectZJ97238 Flavi Shuangao lacewing virus 2
fly11645 Flavi Shayang fly virus 4
SXSSP2490 Flavi Sanxia water strider virus 6
spider134014 Flavi Shayang spider virus 4
spider134009 Flavi Xinzhou spider virus 3
ADK12629 GB virus D
NC 001837 HGvAgp1
fishG49106 Wenling shark virus
YP 009058898 Equine hepacivirus
NC 004102 HCVgp1
Q69422 POLG GBVB
YP 007905733 Rodent hepacivirus
Flavivirus
Jingmenvirus
Pestivirus
Hepacivirus and Pegivirus
1
